# Neuroprotective effects of empagliflozin and metformin combined therapy on rotenone induced Parkinsonism in Wistar rats via PI3K and PPARγ modulation

**DOI:** 10.1038/s41598-026-68289-w

**Published:** 2026-09-08

**Authors:** Marwa M. Mona, Hany M. Borg, Al-Shimaa A. Ahmed, Shimaa A. Abass, Sanad S. El-Kholy

**Affiliations:** 1 Department of Medical Biochemistry and Molecular Biology, Faculty of Medicine, Kafrelsheikh University, Kafrelsheikh, Egypt; 2 Department of Medical Physiology, Faculty of Medicine, Kafrelsheikh University, El-Geish Street, Kafrelsheikh, 33155 Egypt; 3 Department of Biochemistry, Faculty of Pharmacy, Kafrelsheikh University, Kafrelsheikh, Egypt

**Keywords:** Parkinsonism, Neuroprotection, PI3-kinases, Metformin, Empagliflozin, PPAR-γ, Diseases, Drug discovery, Neurology, Neuroscience

## Abstract

Parkinsonism is a progressive neurodegenerative disorder characterized by dopaminergic neuronal loss and α-synuclein aggregation within the substantia nigra pars compacta (SNc). We investigated the potential neuroprotective effects of metformin and empagliflozin, individually and combined, in a rotenone-induced Parkinsonism in rats. Rats were assigned to control, rotenone (ROT), metformin + ROT, empagliflozin + ROT, and combined metformin+empagliflozin + ROT groups. Motor function was assessed using the open field test, static rod test, and inverted grid test. Both metformin and empagliflozin monotherapies significantly improved motor deficits, while the combination therapy demonstrated superior efficacy compared to either treatment alone (*p* < 0.05). Histopathological and immunohistochemical findings further showed that the combined therapy markedly attenuated neuronal degeneration and α-synuclein deposition relative to monotherapies (*p* < 0.001). Biochemically, metformin was more effective than empagliflozin in preserving dopamine levels, with a significant difference observed between the two treatments (*p* < 0.001). Notably, the combination therapy achieved the greatest overall improvement, significantly surpassing both the ROT group and empagliflozin (*p* < 0.001) and showing comparable effects to metformin (*p* > 0.05). Additionally, the combined treatment restored PI3K/AKT signaling activity and significantly upregulated PPAR-γ expression (*p* < 0.001 vs. ROT). Collectively, these findings suggest that combined metformin and empagliflozin therapy exerts enhanced neuroprotective effects through modulation of protein aggregation pathways, highlighting a promising drug-repositioning strategy for Parkinsonism.

**Supplementary Information:**

The online version contains supplementary material available at https://doi.org/10.1038/s41598-026-68289-w.

## Introduction

Parkinsonism is a common neurodegenerative disorder affecting the central nervous system, predominantly in older adults^1,2^. It is characterized by the progressive loss of dopaminergic neurons in the substantia nigra^3^ and the pathological accumulation of misfolded α-synuclein into amyloid fibrils that form Lewy bodies^4^. These inclusions underlie the cardinal motor manifestations of Parkinsonism—rigidity, postural instability, bradykinesia, and resting tremor^5,6^. The neurodegenerative cascade in Parkinsonism arises from multiple interrelated mechanisms, including neuroinflammation, mitochondrial dysfunction, and oxidative stress^2,6^. Rotenone (ROT), a mitochondrial complex I inhibitor, is widely used to reproduce Parkinsonism-like pathology in experimental models. By disrupting the electron transport chain and oxidative phosphorylation, ROT promotes excessive reactive oxygen species (ROS) generation, thereby inducing endoplasmic reticulum (ER) stress, which in turn activates the ER-associated degradation (ERAD) pathway, and ultimately triggers neuronal apoptosis^7,8^.

Approved therapies for Parkinson’s disease include Levodopa/Carbidopa (most effective but causes long-term dyskinesia and fluctuations), Pramipexole and Ropinirole (longer action but less potent and cause impulse-control/psychiatric effects), Rasagiline (well tolerated but modest efficacy), Entacapone (prolongs levodopa but may worsen dyskinesia), and Amantadine (reduces dyskinesia but causes CNS side effects). Overall, these therapies are mainly symptomatic and limited by declining efficacy and adverse effects, highlighting the need for new disease-modifying strategies targeting non-dopaminergic pathways^9–13^.

Epidemiological and experimental evidence increasingly support a link between Parkinsonism (PD) and diabetes mellitus (DM), both of which are highly prevalent and strongly associated with aging populations. These conditions share several underlying pathophysiological mechanisms, including insulin resistance, mitochondrial dysfunction, oxidative stress, chronic inflammation, and disrupted protein homeostasis^14^.

In light of this overlap, and given the growing evidence connecting DM with neurodegeneration, it is reasonable to consider the potential of antidiabetic agents to influence the progression of PD^15^.

Drug repositioning, the strategy of identifying new therapeutic uses for established drugs, has recently gained attention as an efficient approach to novel treatments for neurodegenerative diseases^16^.

Empagliflozin, a sodium-glucose cotransporter-2 (SGLT2) inhibitor, lowers blood glucose by inhibiting glucose reabsorption in the renal proximal tubules and small intestine^17^. In addition to its euglycemic effects, empagliflozin attenuates cognitive deterioration in diabetic patients. The expression of SGLT1 and SGLT2 transporters in the brain suggests a potential role for these proteins in neuroprotection^18^. The neuroprotective actions of empagliflozin have been attributed to activation of the nuclear factor erythroid 2-related factor 2 (Nrf2/Keap1) signaling pathway, thus reinforcement of endogenous antioxidant defenses^8^.

Metformin, a first-line agent for type 2 diabetes mellitus^19^, exerts pleiotropic effects beyond glycemic control. It enhances cellular antioxidant capacity and suppresses inflammation through AMP-activated protein kinase (AMPK) activation. Owing to its lipophilic nature, metformin readily crosses the blood–brain barrier, allowing it to counteract neuronal apoptosis, oxidative injury, mitophagy impairment, and protein aggregation—processes central to neurodegeneration^20^.

Together, these properties suggest that metformin and empagliflozin may confer complementary neuroprotective benefits in Parkinsonism. Recently, several studies have investigated metformin or empagliflozin as monotherapy in experimental models of Parkinsonism. Therefore, in the present study, we intended to investigate the efficacy of combined administration of metformin and empagliflozin as a potential enhanced therapeutic strategy to counteract the neurodegenerative effects of rotenone-induced Parkinsonism in rats.

## Materials and methods

### Ethical statement

All experimental procedures followed the Guide for the Care and Use of Laboratory Animals (U.S. National Institutes of Health, 1986) and were approved by the Institutional Animal Care and Use Committee of Kafrelsheikh University, Egypt (Approval No. KFS-IACUC/150/2023). Experiments were carried out in the Department of Medical Physiology, Faculty of Medicine, Kafrelsheikh University. This study was conducted and reported in accordance with the ARRIVE 2.0 guidelines. (Supplementary File 1)

### Experimental animals

Fifty male Wistar albino rats, aged 6–8 weeks and weighing 180–200 g, were obtained from the Egyptian Organization for Biological Products and Vaccines (Agouza, Giza, Egypt). Animals were accommodated for two weeks before the experiment under standard laboratory conditions: a 12-h light/dark cycle, temperature maintained at 21–25 °C, with relative humidity of 55 ± 5%. Rats were housed in well-ventilated polyethylene cages (floor area: 900 cm²), with 2–3 animals per cage, and had free access to a standard pellet diet and water ad libitum.

### Drugs, chemicals, and kits

The drugs used in the present study included ROT, empagliflozin, and metformin. ROT was obtained from Sigma-Aldrich (St. Louis, MO, USA; Cat. No.: R8875). Empagliflozin was provided in the form of Empacoza tablets containing 10 mg per tablet (ZETA Pharma, Cairo, Egypt). Metformin was supplied as Glucophage XR tablets containing 1000 mg per tablet (MERCK Co., Darmstadt, Germany).

The primary antibodies used in this study included PI3 Kinase p85 (Cat. No.: #4292; Cell Signaling Technology, RRID: AB_659940, Danvers, MA, USA) and Phospho-PI3 Kinase p85 (Tyr458)/p55 (Tyr199) (Cat. No.: #4228; Cell Signaling Technology, RRID: AB_659938, Danvers, MA, USA). β-actin antibody (Cat. No.: ab8226; RRID: AB_306371, Abcam, Cambridge, UK) was employed as a loading control. Additionally, TriFast reagent (Peqlab, VWR International, Erlangen, Germany; Cat. No.: 30-2010) was utilized for the simultaneous isolation of RNA, DNA, and protein.

The High Pure RNA Isolation Kit (Cat. No. 11 828 665 001; Roche Diagnostics GmbH, Germany) was utilized for the extraction of total RNA. Striatal dopamine concentrations were quantified using the Elabscience^®^ Dopamine (DA) ELISA Kit (Cat. No.: E-EL-0046; Elabscience, Houston, TX, USA).

### Drug preparation

ROT was prepared at a dose of 3 mg/kg by dissolving 20 mg in 20 mL of sunflower oil to obtain a final concentration of 1 mg/mL, followed by sonication for 10 min to ensure uniform dispersion^21^. The ROT dose (3 mg/kg/day) and duration of administration (10 days) were selected based on previously established experimental protocols that induce reproducible Parkinsonism-like features within a subacute timeframe. This regimen has been reported to produce significant dopaminergic neuronal dysfunction, oxidative stress, and behavioral impairments while maintaining acceptable tolerability when animals are carefully monitored. The present study was designed to model early-stage neurodegenerative changes, allowing the evaluation of therapeutic interventions on initial pathogenic mechanisms rather than late-stage chronic progression. Animals were monitored daily for signs of toxicity, and no excessive mortality was observed during the experimental period^22^.

Empagliflozin was administered at a dose of 10 mg/kg and prepared by finely grinding one Empacoza tablet (25 mg) and suspending it in 10 mL of distilled water to yield a final concentration of 2.5 mg/mL^5^. Metformin was prepared at a dose of 150 mg/kg by grinding one Glucophage XR tablet (1000 mg) and suspending it in 20 mL of distilled water to achieve a final concentration of 50 mg/mL^2^.

### Experimental design and sample size

**Fig. 1:**
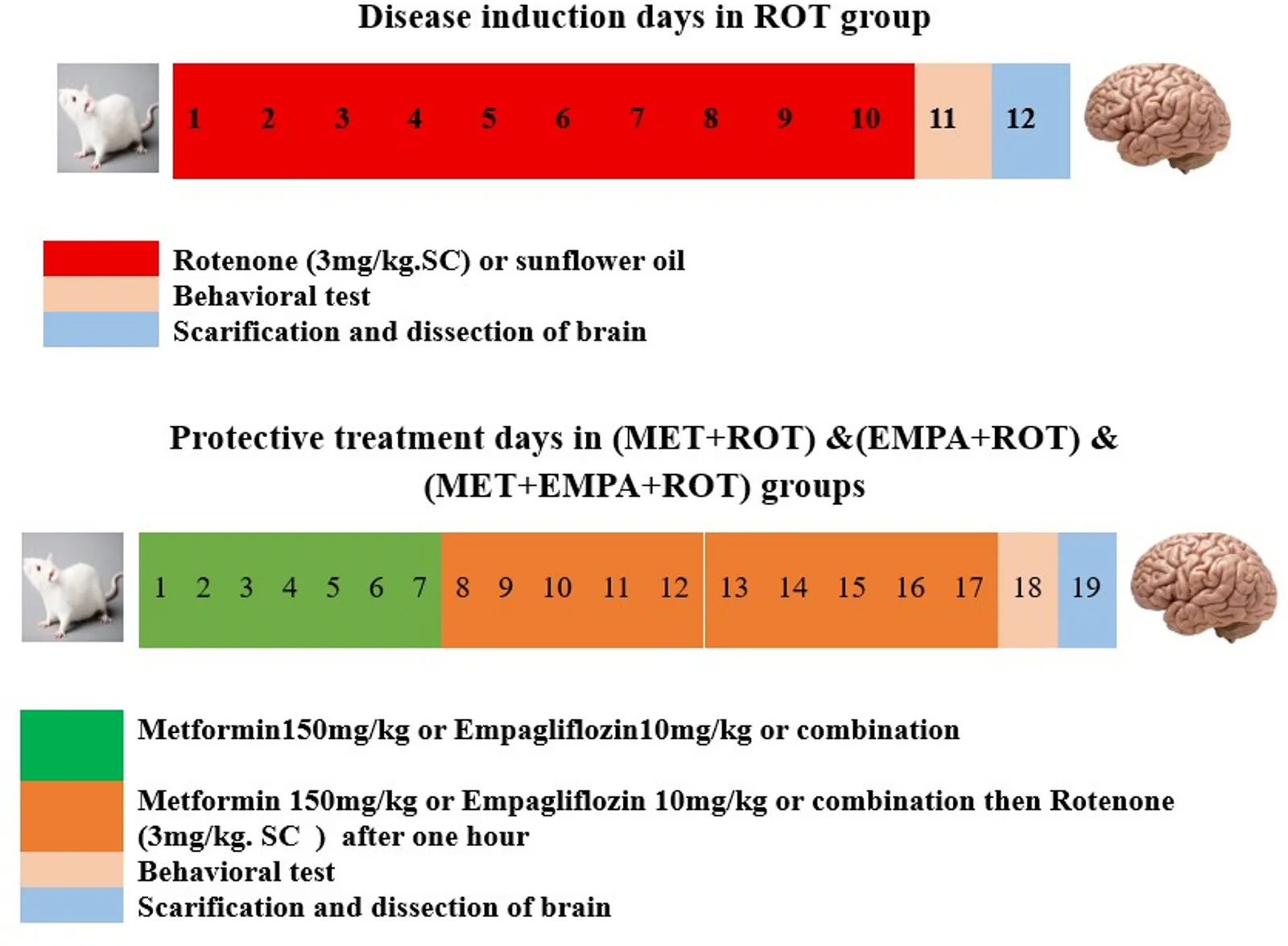
Schematic representation of the experimental study design illustrating group allocation, rotenone (ROT)-induced Parkinsonism model induction, and treatment regimens including metformin (MET), empagliflozin (EMPA), and their combination *(n = 10 per group)*.

After an acclimatization period, the rats were randomly allocated into five groups (*n* = 10 per group). The Control group received subcutaneous injections of sunflower oil (vehicle) once daily for ten consecutive days, along with oral administration of distilled water on the corresponding days, similar to the preventive treatment schedule of the other groups. The ROT group was administered ROT at a dose of 3 mg/kg/day subcutaneously for 10 days. The Metformin + Rotenone (MET + ROT) group received oral pretreatment with metformin (150 mg/kg/day) for 7 days before rotenone exposure, followed by co-administration for 10 days. The Empagliflozin + Rotenone (EMPA + ROT) group was pretreated with empagliflozin (10 mg/kg/day, oral) for 7 days prior to ROT exposure, then co-treated for an additional 10 days. Finally, the Metformin + Empagliflozin + Rotenone (MET + EMPA + ROT) group received both metformin and empagliflozin at the same respective doses for 7 days before ROT exposure, followed by combined treatment with ROT for 10 days (Fig. 1).

Rats were randomly assigned to the experimental groups using a computer-generated random number sequence generated with Random Allocation Software, Version 2.0 (developed by Mahmood Saghaei, Isfahan University of Medical Sciences, Iran; https://random-allocation-software.software.informer.com/2.0/). The study was conducted using a single-blinded design, in which all outcome assessments—including behavioral evaluations, biochemical analyses, and histopathological examinations—were performed by investigators blinded to the treatment group allocation. This procedure was implemented to minimize observer bias and to improve the validity, reliability, and objectivity of the experimental findings.

The sample size (*n* = 50; 10 animals per group) was determined based on previous studies employing rotenone-induced Parkinsonism models and was further supported by an a priori power analysis performed using GPower software (version 3.1.9.7). A one-way ANOVA (fixed effects, omnibus) was selected as the statistical test, with an assumed large effect size (Cohen’s f = 0.40), significance level α = 0.05, and statistical power (1 − β) = 0.80. According to the GPower calculation, the minimum required sample size was approximately 10 animals per group to detect statistically significant differences among the experimental groups while maintaining compliance with ethical principles for animal use and reduction. The selected effect size was based on previously published studies of rotenone-induced Parkinsonism, which demonstrated substantial behavioral, biochemical, and histopathological differences between experimental groups^6,23,24^.

### Behavioral assessment

Behavioral testing was performed one day after the final treatment using three validated paradigms to evaluate locomotor, motor coordination, and muscle strength parameters. All behavioral tests were performed under identical environmental conditions and at approximately the same time of day to minimize circadian variation. Each animal was tested individually, and the same apparatus and software settings were used throughout the study. Behavioral data obtained from each animal were considered a single biological replicate for statistical analysis. No animals were excluded from the analyses, and no outlier exclusion criteria were applied.

#### Open field test

The open field apparatus consisted of a wooden arena measuring 100 × 100 cm with walls 50 cm high, divided into 16 equal squares. Behavioral activity was recorded using an overhead digital camera and analyzed with ANY-maze software (Stoelting Co., USA). Each rat was placed in one corner of the arena and allowed to freely explore for 5 min. To eliminate odor cues, the arena was thoroughly cleaned with 70% ethanol between trials^25^. The parameters measured included total distance traveled (m), average movement speed (m/s), number of line crossings, and frequency of rearing and grooming behaviors. Software calibration, tracking thresholds, and detection parameters were standardized across all animals to ensure consistency^25^. (Supplementary Video 1)

#### Static rod test

Motor coordination and balance were assessed according to the method described by Deacon (2013)^26^. Two horizontal wooden rods, measuring 5 cm and 1.5 cm in diameter, were positioned 60 cm above the floor. Each rat was placed on the distal end of the rod facing away from the shelf. The measured parameters included orientation time, defined as the time taken to turn 180° toward the shelf, and Transit Time – the duration taken by the rat to move forward until its nose crossed the 10 cm marker closest to the shelf. The maximum orientation time was limited to 120 s^26^ (Supplementary Video 2).

#### Inverted grid test

This test assessed grip strength and balance^27^. Rats were placed on a 50 × 50 cm wire mesh grid (0.5 cm² openings). The grid was inverted, and the latency to fall was recorded. Each rat performed 3 trials separated by 3-min intervals; the longest hanging time was recorded^27^. (Supplementary Video 3)

### Tissue collection (sampling)

Following the completion of behavioral testing (on experimental day 19, 24 h after the last test), the rats were anesthetized with pentobarbital sodium (200 mg/kg, i.p.), an AVMA-approved humane euthanasia method that ensures rapid unconsciousness while minimizing physiological stress that could confound downstream molecular analyses, and subsequently euthanized by cervical dislocation performed by trained personnel to ensure complete and ethical termination^28^.

The brains were rapidly excised, rinsed with saline under aseptic conditions, gently blotted dry, and weighed before further processing. Each brain was then divided into two hemispheres. One hemisphere was fixed in 10% neutral-buffered formalin (pH 7.4) for histopathological examination and immunohistochemical analysis of α-synuclein. The second hemisphere was further subdivided and stored at − 80 °C for subsequent biochemical analyses.

### Histopathological and immunohistochemical examination

#### Histopathological analysis

Following fixation, tissues were processed for paraffin embedding, sectioned at 5 μm, and stained with hematoxylin and eosin (H&E) to evaluate histopathological alterations under light microscopy. Sections were examined using a bright-field light microscope (Olympus CX21, Tokyo, Japan) equipped with an Olympus DP21 digital camera. Images were captured using 4× (NA 0.10), 10× (NA 0.25), 20× (NA 0.40), and 40× (NA 0.65) objective lenses, with corresponding final magnifications of 40×, 100×, 200×, and 400×, respectively. All images used for quantification of α-synuclein immunoreactivity were acquired at 400× magnification (40× objective with 10× eyepiece) under consistent illumination settings across all experimental groups. For each animal, one representative section was analyzed, and three non-overlapping photomicrographs were captured per section at standardized magnifications for evaluation. Degenerated neurons were identified according to established morphological criteria, including shrunken cell bodies, intensely eosinophilic cytoplasm, pyknotic or condensed nuclei, and disruption of normal neuronal architecture. Degenerated neurons were manually counted by a blinded observer using ImageJ, Version 1.54 (National Institutes of Health, NIH, USA; https://imagej.net/ij/), and the mean number of degenerated neurons per microscopic field was calculated for statistical analysis^29,30^ (Supplementary File 2).

#### Immunohistochemical detection of α-synuclein aggregates

α-Synuclein immunohistochemistry was performed using a rabbit polyclonal anti-α-synuclein antibody (1:2000; GeneTex, USA), with the cerebellum serving as a positive control. Paraffin-embedded brain sections were deparaffinized, rehydrated, blocked with 5% normal goat serum and 0.3% Triton X-100, and incubated overnight with the primary antibody at 4 °C. After washing, sections were incubated with a biotinylated goat anti-rabbit IgG secondary antibody (1:200). Signal amplification was performed using the avidin–biotin complex (ABC) system (Vector Laboratories), prepared freshly according to the manufacturer’s instructions and incubated for 30 min at room temperature under standardized conditions for all samples. Immunoreactivity was visualized using 3,3′-diaminobenzidine (DAB), prepared as a freshly made working solution following the manufacturer’s protocol, and developed uniformly across all sections. Staining reactions were stopped once optimal signal intensity was achieved. Sections were examined using bright-field microscopy, and α-synuclein immunoreactivity in the substantia nigra and striatum was quantified using ImageJ software as percentage positive area and integrated optical density (IOD)^31^.

### Biochemical analysis

#### Determination of striatal dopamine by ELISA

Striatal dopamine levels were quantified using the Elabscience^®^ Dopamine ELISA Kit (Cat. No. E-EL-0046; Elabscience, China) following the manufacturer’s protocol. Absorbance was measured with a HiPo MPP-96 Microplate Photometer at a wavelength of 450 nm, and dopamine concentrations were determined based on the standard calibration curve. All measurements were performed using a commercially validated ELISA kit, and samples were analyzed according to the manufacturer’s instructions. A standard calibration curve was generated for each assay run using known dopamine concentrations to ensure linearity and accurate quantification. Intra-assay variability was minimized by running all samples in duplicate within the same plate, and inter-assay consistency was ensured by performing all experimental groups under identical assay conditions. Dopamine levels were normalized to total protein concentration in brain homogenates to account for variability in tissue input^32^.

#### Western blotting

Total protein was extracted using TriFast reagent (a monophasic solution for simultaneous isolation of RNA, DNA, and proteins), quantified by the Bradford assay^33^, and separated by SDS–PAGE using 10% resolving and 4% stacking gels^34^. For each sample, 30 µg of protein were loaded onto the gel. Electrophoresis was carried out at 75 V through the stacking gel, followed by 125 V through the resolving gel for approximately 2 h. Subsequently, proteins were transferred onto Hybond™ nylon membranes (GE Healthcare).

The membranes were blocked with 5% non-fat milk in Tris-buffered saline containing 0.1% Tween-20 (TBST) and then incubated overnight at 4 °C with primary antibodies against PI3K p85 (#4292; dilution 1:1000, Cell Signaling Technology) or phospho-PI3K p85 (Tyr458)/p55 (Tyr199) (#4228; dilution 1:1000, Cell Signaling Technology). After washing, membranes were incubated with horseradish peroxidase (HRP)-conjugated secondary antibodies for 1 h at room temperature. β-Actin (ab8226) was used as a loading control. Immunoreactive bands were visualized using a GelDoc-It Imaging System (UVP, UK) under exposure conditions kept within the linear detection range to avoid signal saturation. Exposure time was optimized per blot and kept consistent within each experimental set. Densitometric analysis was performed using TotalLab software (v1.0.1) with identical background subtraction settings across all samples^31–33,35^.For Western blot analysis, three animals were randomly selected from each experimental group (Supplementary File 3).

#### Quantitative PCR analysis

Total RNA was extracted using the High Pure RNA Isolation Kit (Roche Diagnostics, Basel, Switzerland). One microgram of total RNA was reverse-transcribed into single-stranded cDNA using the QuantiTect^®^ Reverse Transcription Kit (Qiagen, USA) following elimination of genomic DNA contamination with gDNA Wipeout buffer. After brief incubation for gDNA removal, first-strand cDNA synthesis was carried out at 42 °C, and reverse transcriptase was subsequently inactivated at 95 °C. Quantitative real-time PCR was performed using the Rotor-Gene Q system (Qiagen, USA) with Maxima^®^ SYBR Green/Fluorescein qPCR Master Mix (Thermo Scientific, USA). Each 25 µL reaction contained 30 ng of cDNA and gene-specific primers (300 nM). PPAR-γ expression was amplified using the forward primer 5′-CATGACCAGGGAGTTCCTCAA-3′ (Thermo Scientific, USA) and the reverse primer 5′-AGCAAACTCAAACTTAGGCTCCAT-3′ (Thermo Scientific, USA). GAPDH was used as the housekeeping gene with the forward primer 5′-CAAGTTCAACGGCACAGTCAAG-3′ (Thermo Scientific, USA) and the reverse primer 5′-CTCCTGGAAGATGGTGATTGGT-3′ (Thermo Scientific, USA). All reactions were performed in duplicate under defined thermal cycling conditions, and threshold cycle (Ct) values were automatically recorded. Relative gene expression levels were calculated using the 2^−ΔΔCt method after normalization to GAPDH. Consistency of amplification was ensured by maintaining identical reaction conditions across all groups^36,37^.

### Data and statistical analysis

All experimental data were expressed as mean ± standard deviation (SD). Statistical analyses were performed using GraphPad Prism software (version 8.0; GraphPad Software Inc., San Diego, CA, USA). The normality of the data distribution was first verified using the Shapiro–Wilk test, while homogeneity of variance was evaluated using Levene’s test prior to parametric analysis. Intergroup comparisons were performed using one-way analysis of variance (ANOVA) followed by Tukey’s Honestly Significant Difference (HSD) post hoc test for multiple comparisons. In cases where these assumptions were not met, the non-parametric Kruskal–Wallis test followed by Dunn’s multiple comparison test was applied. To minimize the risk of Type I error associated with multiple comparisons across behavioral and biochemical endpoints, appropriate post hoc correction procedures (Tukey’s or Dunn’s tests, as applicable) were used. Differences were considered statistically significant at *p* ≤ 0.05 and highly significant at *p* < 0.001.

## Result

### Neurobehavioral assessment

#### Open field test

**Fig. 2:**
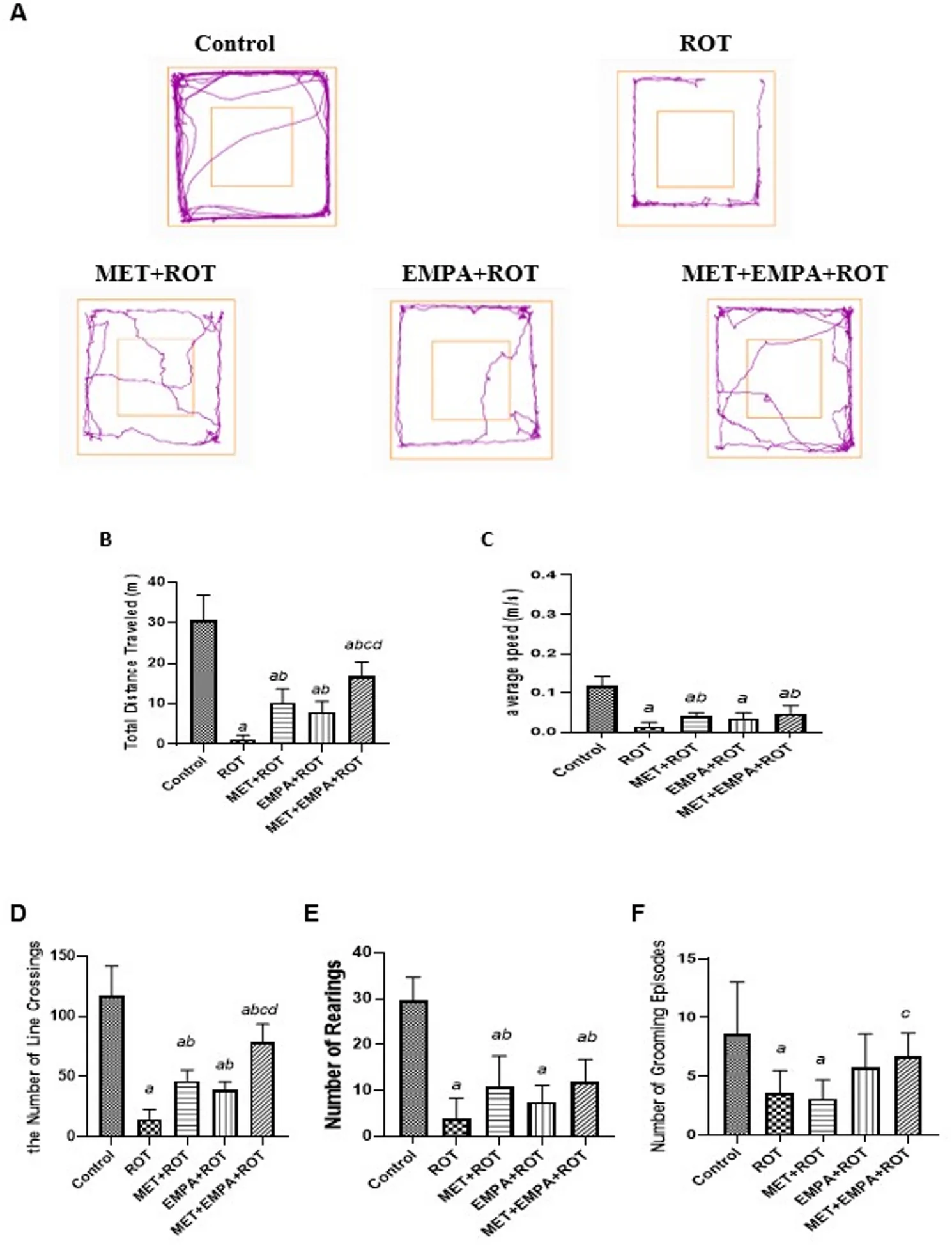
(**A**) Representative locomotor activity tracks of rats from five experimental groups during the Open Field Test. Movement paths (purple) recorded continuously using the ANY-maze video tracking system. The outer square denotes the arena boundary; the inner square indicates the central zone for center vs. periphery analysis *(n = 10 per group)*. (**B**) Total distance traveled (m) in the Open Field Test across groups (Control, ROT, MET + ROT, EMPA + ROT, MET+EMPA + ROT). Data presented as mean ± SD. Data are presented as mean ± standard deviation (SD). Statistical significance: (a) vs. Control group; (b) vs. ROT group; (c) vs. MET + ROT group; (d) vs. EMPA + ROT group. Analyses were performed using one-way ANOVA followed by Tukey’s post hoc test or Kruskal–Wallis test with Dunn’s correction, as appropriate *(n = 10 per group)*. (**C**) Average speed (m/s) in the Open Field Test; notation as in (B). (**D**) Number of line crossings; data and significance as in (B). (**E**) Number of rearings; data and significance as in (B). (**F**) Number of grooming episodes; data and significance as in (B).

The ROT group showed a marked reduction in total distance traveled (1.36 ± 0.96 m) compared with controls (30.70 ± 6.18 m; *P* < 0.001), confirming successful induction of Parkinsonian-like motor deficits. Both metformin (MET + ROT, 10.24 ± 3.44 m) and empagliflozin (EMPA + ROT, 7.88 ± 2.82 m) significantly increased total distance compared with the ROT group (*P* < 0.001 and *P* < 0.05, respectively), with no significant difference between monotherapies. The combined treatment (MET + EMPA + ROT, 14.87 ± 4.50 m) produced the highest recovery, significantly exceeding both single-drug groups (*P* < 0.05) and the ROT group (*P* < 0.001) (Fig. 2).

Average locomotor speed was also significantly decreased by ROT (0.014 ± 0.010 m/s vs. 0.117 ± 0.024 m/s; *P* < 0.001). Metformin and empagliflozin improved this parameter (0.0396 ± 0.009 m/s and 0.0349 ± 0.014 m/s, respectively; *P* < 0.05 vs. ROT), though not to control levels. Importantly, no significant difference was observed between the metformin and empagliflozin treatment groups (P3 and P4: *P* > 0.05), suggesting similar efficacy in mitigating motor decline. Combined treatment further improved speed (0.0468 ± 0.021 m/s), significantly different from the ROT group (*P* < 0.001). The number of line crossings, reflecting horizontal exploration, decreased sharply in the ROT group (14.7 ± 8.10) compared with controls (117.4 ± 24.5; *P* < 0.001). Metformin and empagliflozin increased crossings (46.5 ± 9.12 and 39.1 ± 6.69, respectively; *P* < 0.001 and *P* < 0.05), with no difference between them. The combination regimen yielded the greatest improvement (78.6 ± 15.2; *P* < 0.001 vs. ROT and monotherapies) (Fig. 2).

Rearing frequency, indicating vertical exploration and motor coordination, was markedly reduced in the ROT group (3.80 ± 4.57 vs. 29.6 ± 5.13; *P* < 0.001). Metformin partially improved this measure (10.8 ± 6.73; *P* < 0.05 vs. ROT), while empagliflozin showed a modest, nonsignificant effect (7.50 ± 3.63). The combined treatment achieved the highest value (12.0 ± 4.78; *P* < 0.05 vs. ROT). Grooming behavior, which reflects emotional and motor activity, was significantly reduced by ROT (3.60 ± 1.90 vs. 8.60 ± 4.43; *P* < 0.001). Metformin showed no improvement (3.10 ± 1.60), while empagliflozin exhibited a slight nonsignificant increase (5.80 ± 2.82). The combined regimen (6.70 ± 2.00) produced the highest mean grooming frequency among treated groups, significantly exceeding metformin alone (*P* < 0.05) (Fig. 2).

#### Motor coordination and balance assessment (static rod test)

**Fig. 3:**
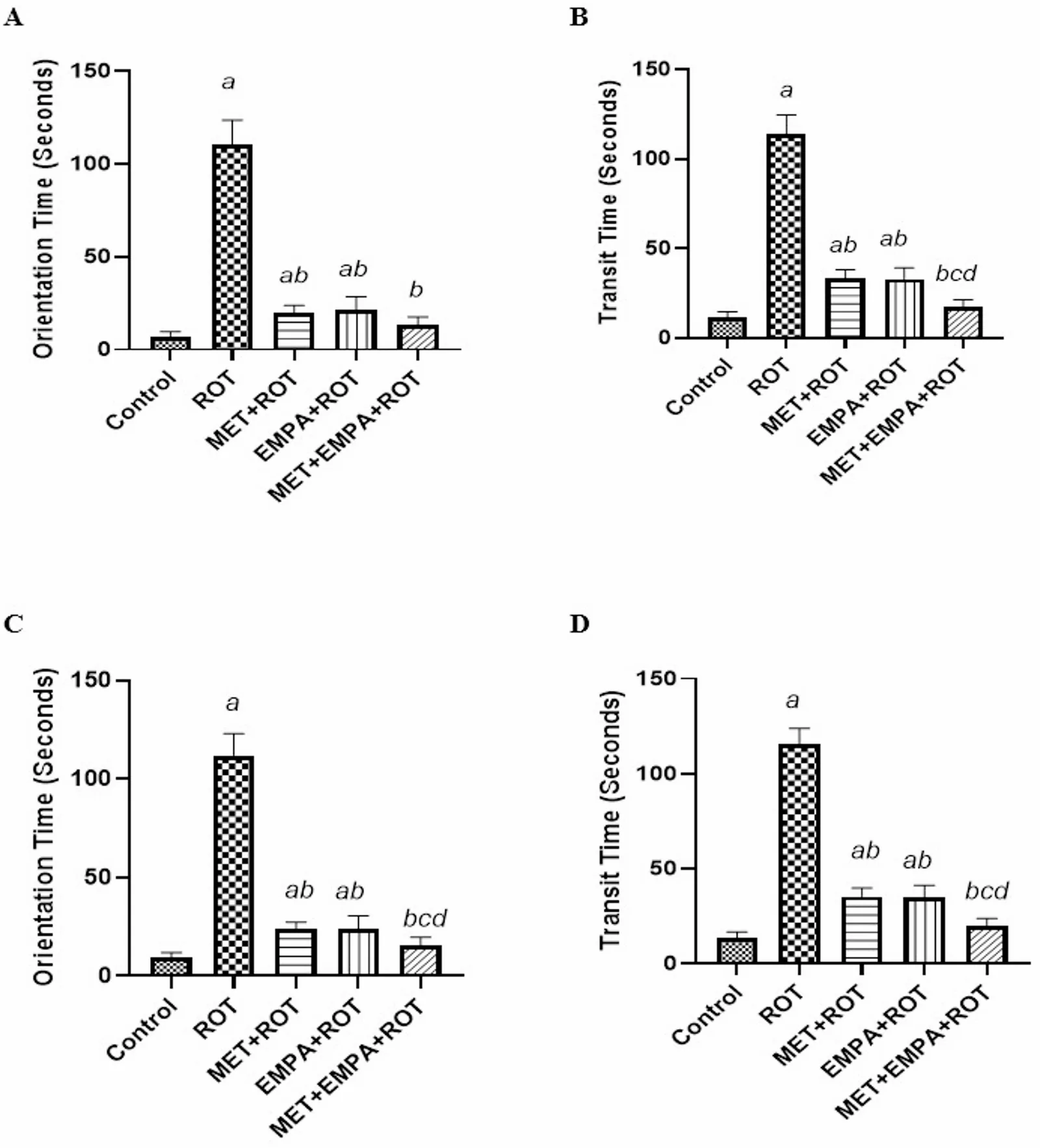
(**A**) Orientation time (seconds) on a 5 cm thick rod in Static Rod Test across groups; data mean ± SD, significance as in (Fig. 2B) *(n = 10 per group)*. (**B**) Transit time (seconds) on 5 cm thick rod; notation as in (A). (**C**) Orientation time on a 1.5 cm thick rod; data and significance as in (A). (**D**) Transit time on 1.5 cm rod; data and significance as in (A).

On a rod with 5 cm thickness, ROT markedly increased orientation time (110.1 ± 13.5 s) compared to controls (6.90 ± 2.69 s; *P* < 0.001). Both metformin and empagliflozin groups significantly shortened this duration (19.9 ± 3.78 s and 21.6 ± 6.90 s, respectively; *P* < 0.001 vs. ROT). No significant difference was observed between the metformin+rotenone and empagliflozin+rotenone groups (*P* > 0.05), whereas combined therapy produced the greatest improvement (13.3 ± 4.22 s; *P* < 0.05 vs. EMPA + ROT), indicating a superior effect of the combination treatment. Transit time exhibited a similar pattern. ROT significantly increased transit time (114.0 ± 10.8 s) compared with controls (11.7 ± 2.83 s; *P* < 0.001). Treatment with metformin or empagliflozin significantly improved this parameter (33.3 ± 4.81 s and 32.9 ± 6.28 s, respectively; *P* < 0.001 vs. ROT), whereas combined therapy significantly produced the most pronounced reduction (17.6 ± 3.89 s; *P* < 0.001 vs. ROT and monotherapies) (Fig. 3).

On the narrower rod (1.5 cm), the ROT group showed a dramatic increase in orientation time (111.9 ± 11.0 s vs. 9.20 ± 2.57 s; *P* < 0.001). Both metformin (24.2 ± 3.12 s) and empagliflozin (23.9 ± 6.54 s) reduced times significantly (*P* < 0.001 vs. ROT), though no significant difference was observed between the two treatments (P3: *P* > 0.05). The combined treatment (15.5 ± 4.06 s) further improved performance (*P* < 0.05 vs. monotherapies). Likewise, transit time on the 1.5 cm rod was markedly prolonged in the ROT group (115.5 ± 8.32 s vs. 13.6 ± 2.91 s; *P* < 0.001). Metformin and empagliflozin shortened the time significantly (35.2 ± 4.44 s and 35.0 ± 6.20 s; *P* < 0.001 vs. ROT), but the difference between the two treatments was not statistically significant (P3: *P* > 0.05). The combined treatment (19.9 ± 3.76 s) resulted in the greatest improvement (*P* < 0.05 vs. monotherapies) (Fig. 3).

Overall, metformin and empagliflozin monotherapies produced modest yet statistically significant improvements in rotenone-induced impairments of balance and motor coordination. In contrast, the combined therapy exerted an enhanced effect, yielding the greatest functional recovery compared with both the diseased group and either monotherapy alone.

#### Muscle strength and grip performance (inverted grid test)

The treatment with metformin or empagliflozin partially reversed this effect, significantly prolonging fall latency compared with the ROT group (*P* < 0.001), although values remained below control levels. Notably, the combined regimen produced the most robust improvement, with fall latency significantly higher than in both the ROT and monotherapy groups (*P* < 0.001) and approaching control values. No significant difference was observed between the MET + ROT and EMPA + ROT groups (*P* > 0.05), indicating comparable efficacy when administered individually (Table 1).

**Table 1:** Comparison of time to fall (seconds) in the inverted grid test across the five groups.

	Control	ROT	MET + ROT	EMPA + ROT	MET+EMPA + ROT
Time to fall (seconds) in the inverted grid test Mean ± S.D	155.5 ± 12.59	9.400 ± 3.169	37.20 ± 8.257	32.40 ± 8.909	95.60 ± 10.94
P1 P2 P3 P4		*P* < 0.001	*P* < 0.001 *P* < 0.001	*P* < 0.001 *P* < 0.001 *P* > 0.05	*P* < 0.001 *P* < 0.001 *P* < 0.001 *P* < 0.001

Each group consisted of 10 rats (*n* = 10). P1: comparison with the control group; P2: comparison with the diseased (ROT) group; P3: comparison with the MET + ROT group; P4: comparison with the EMPA + ROT group. A P-value ≤ 0.05 was considered statistically significant, while *P* ≤ 0.001 was considered highly significant. Data are expressed as mean ± standard deviation (SD).

### Immunohistochemical and histopathological findings

Hematoxylin and eosin (H&E) staining demonstrated a marked increase in the number of degenerated neurons in the substantia nigra pars compacta (SNc) of the ROT group (75.52 ± 2.925) compared with the control group (4.050 ± 0.9419), confirming severe ROT-induced neurodegeneration (*P* < 0.001). Treatment with metformin and empagliflozin significantly attenuated neuronal degeneration, with mean counts of 25.59 ± 1.849 and 38.59 ± 2.451, respectively (*P* < 0.001), indicating partial neuroprotection. The combination therapy exerted the greatest protective effect, reducing the mean degenerated neuronal count to 10.27 ± 1.120, which was significantly lower than those observed with either monotherapy (*P* < 0.001) and closely approximated control values.

**Fig. 4:**
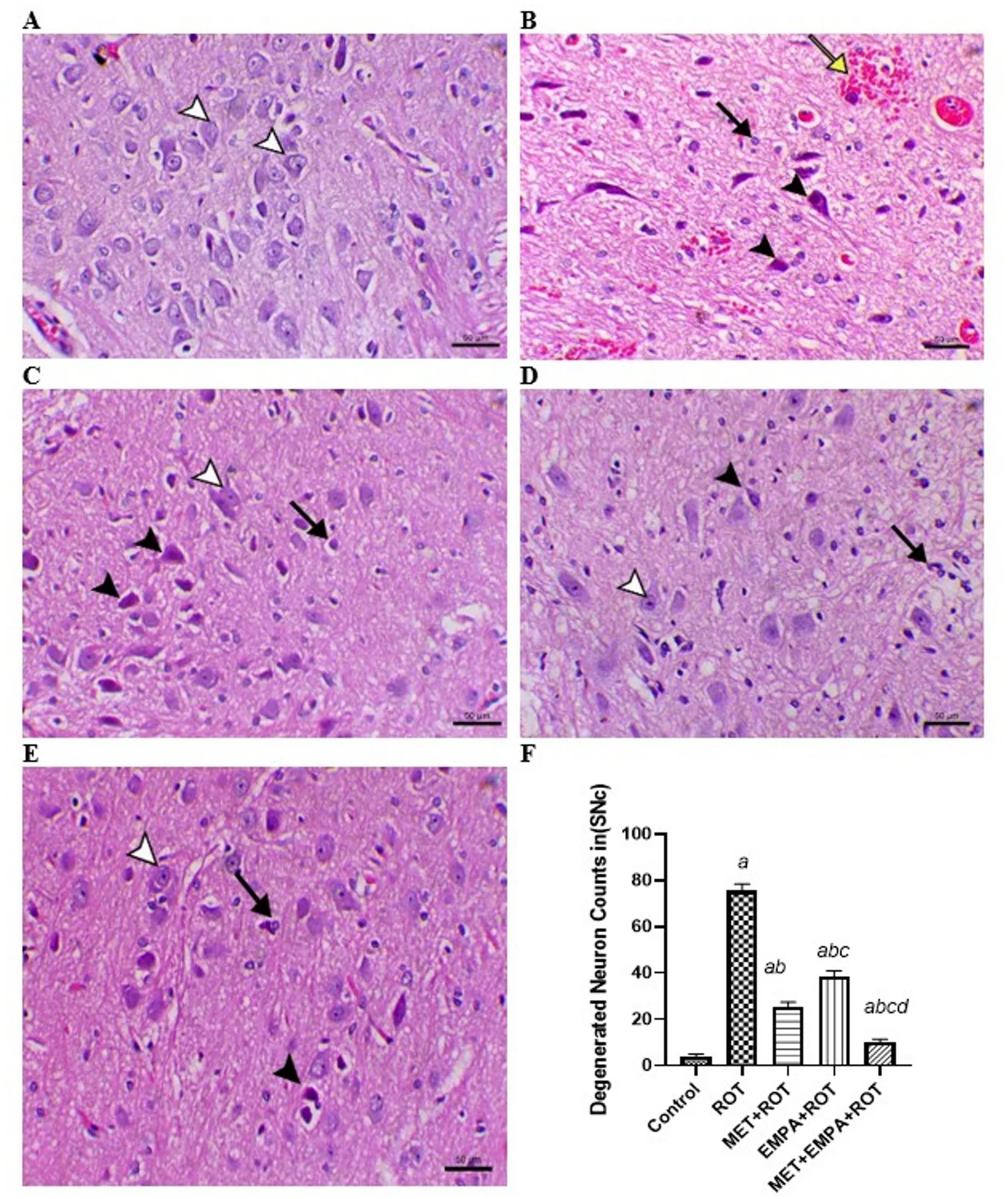
(**A**) Substantia nigra pars compacta of control (G1) showing normal neurons (arrowheads); Hematoxylin and eosin (H&E) stain, scale bar = 50 μm. (**B**) Diseased (G2) showing severe ischemic injury, gliosis (black arrow), and hemorrhage (yellow arrow); H&E stain. (**C**) Metformin-treated (G3) showing reduced ischemia, vacuolation, decreased gliosis/neuronophagia; H&E stain. (**D**) Empagliflozin-treated (G4) showing reduced ischemic features and mild gliosis; H&E stain. (**E**) Combined treatment (G5) showing marked ischemia reduction and increased normal neurons; H&E stain. (**F**) Quantification of degenerated neurons in the substantia nigra pars compacta. Data are expressed as mean ± SD with statistical significance as in (Fig. 2B) (*n* = 10 per group).

**Fig. 5:**
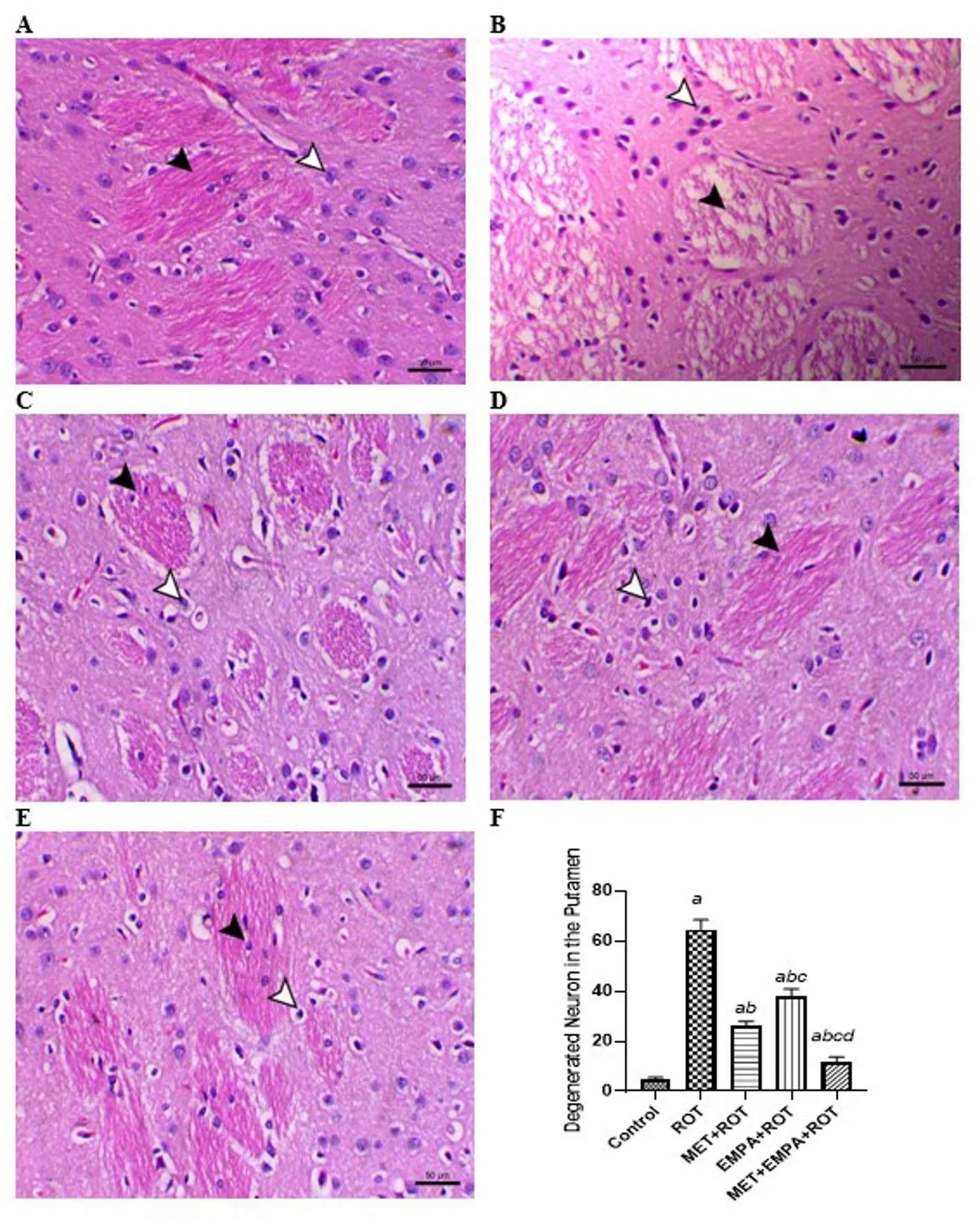
(**A**) Putamen from control (G1) showing normal neurons and striatal fibers; H&E stain, scale bar = 50 μm. (**B**) Diseased (G2) showing nuclear pyknosis and fiber disruption; H&E stain. (**C**) Metformin-treated (G3) showing perineuronal edema and vacuolation; H&E stain. (**D**) Empagliflozin-treated (G4) showing decreased basophilic neurons, preserved fibers; H&E stain. (**E**) Combined treatment (G5) with mild vacuolation, preserved fibers; H&E stain. (**F**) Quantification of degenerated neurons in the putamen.

Similarly, H&E staining of the putamen revealed a substantial increase in degenerated neuronal counts in the ROT group (64.67 ± 3.930) compared with the control group (4.715 ± 0.8566), indicating pronounced ROT-induced neurotoxicity (*P* < 0.001). Treatment with metformin (26.26 ± 1.663) and empagliflozin (38.13 ± 2.805) significantly reduced neuronal degeneration (*P* < 0.001), with metformin demonstrating greater efficacy than empagliflozin (*P* < 0.001). The combination therapy provided the most substantial neuroprotection (11.82 ± 1.844), significantly surpassing the effects of both monotherapies (*P* < 0.001) (Figs. 4 and 5).

Immunohistochemical analysis of the substantia nigra pars compacta (SNc) demonstrated a marked increase in the alpha-synuclein–positive area in the ROT group (57.17 ± 3.236) compared with the control group (4.662 ± 0.8207), confirming significant pathological accumulation of alpha-synuclein following ROT exposure (*P* < 0.001). Treatment with metformin (21.87 ± 2.165) and empagliflozin (31.23 ± 2.118) significantly reduced alpha-synuclein burden (*P* < 0.001), with metformin exerting a more pronounced effect than empagliflozin (*P* < 0.001). The combination therapy achieved the greatest reduction (8.436 ± 0.7577), approaching control values and significantly exceeding the effects of both monotherapies (*P* < 0.001).

**Fig. 6:**
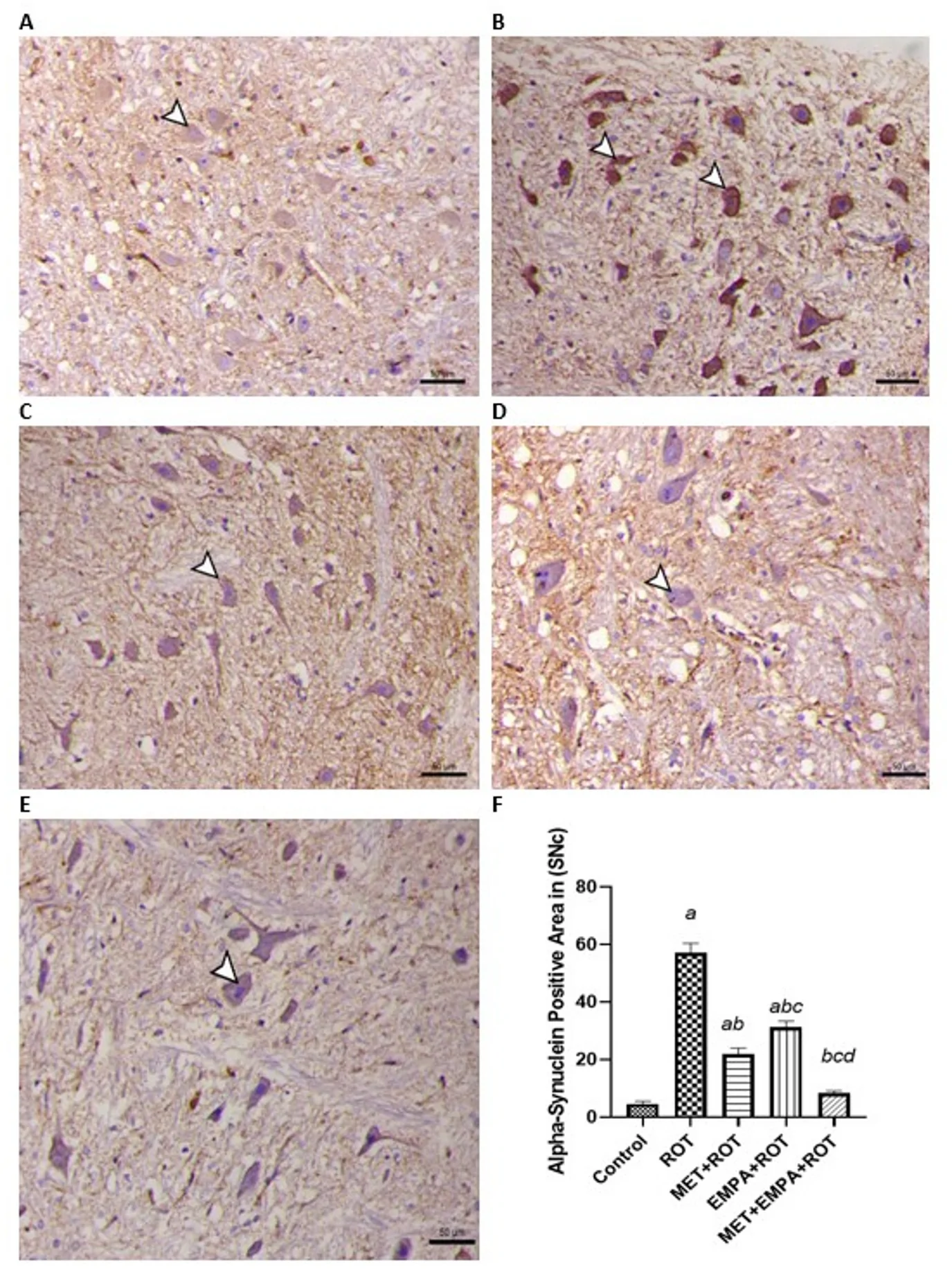
Alpha-synuclein immunohistochemistry in substantia nigra pars compacta: Scale bar = 50 μm. (**A**) Control (G1) with mild cytoplasmic α-synuclein in neurons (arrowhead). (**B**) Diseased (G2) with marked α-synuclein expression (arrowheads). (**C**) Metformin-treated (G3) showing decreased α-synuclein (arrowhead). (**D**) Empagliflozin-treated (G4) with reduced expression (arrowheads). (**E**) Combined treatment (G5) with marked decrease in cytoplasmic α-synuclein (arrowhead). (**F**) Quantification of α-synuclein–positive area (%) within 1 mm² SNc. Data are expressed as mean ± SD with statistical significance as in (Fig. 2B) (*n* = 10 per group).

**Fig. 7:**
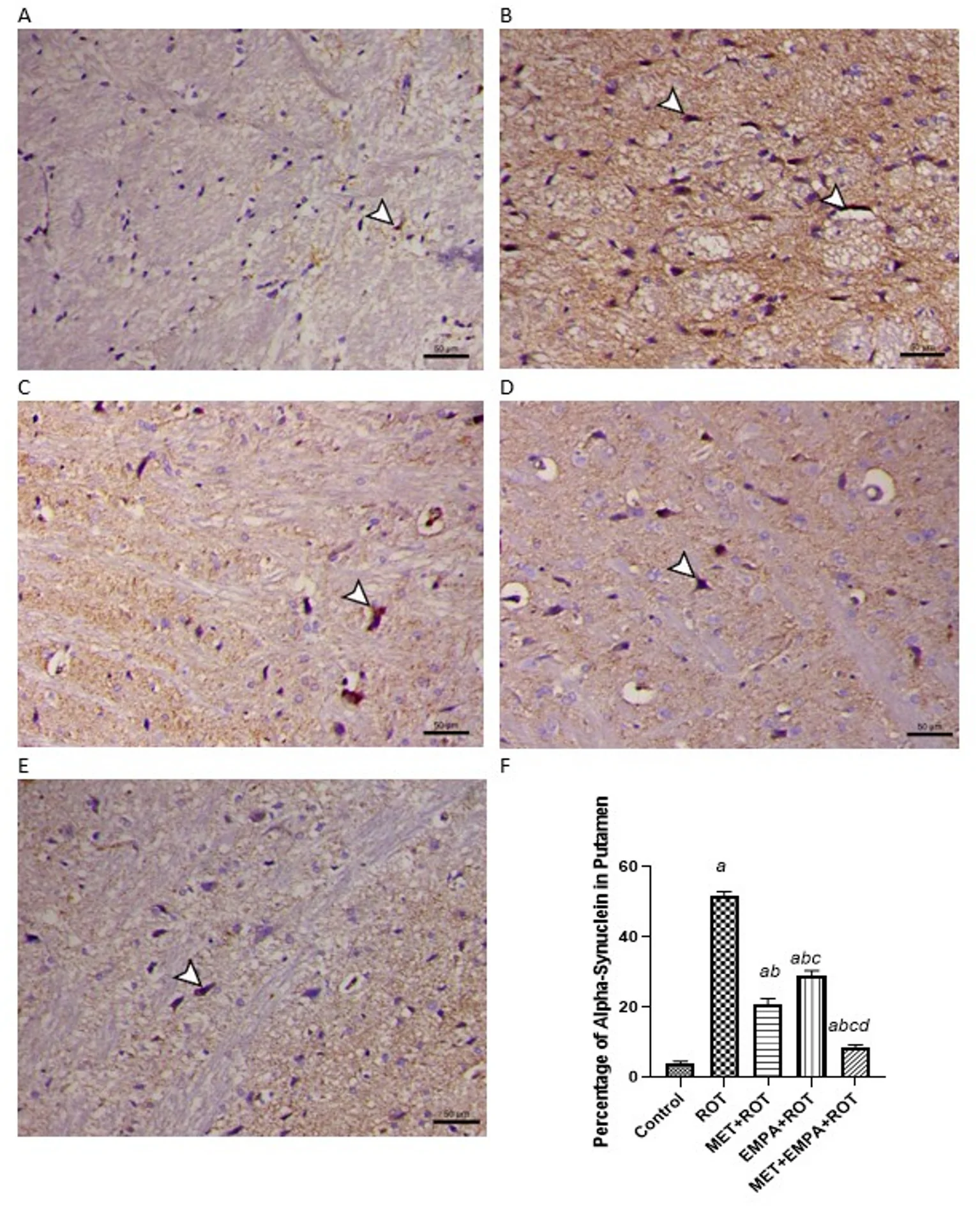
Alpha-synuclein immunohistochemistry in the putamen region: Scale bar = 50 μm. (**A**) Control (G1) mild α-synuclein in neurons (arrowhead). (**B**) Diseased (G2) increased expression (arrowheads). (**C**) Metformin-treated (G3) decreased expression (arrowhead). (**D**) Empagliflozin-treated (G4) reduced expression (arrowhead). (**E**) Combined treatment (G5) marked decrease in expression (arrowhead). (**F**) Quantification of α-synuclein–positive area (%) within 1 mm² putamen. Data are expressed as mean ± SD with statistical significance as in (Fig. 2B) (*n* = 10 per group).

Likewise, immunohistochemical assessment of the putamen revealed a significant increase in the alpha-synuclein–positive area in the ROT group (51.64 ± 1.393) compared with the control group (3.940 ± 0.6860), indicating marked ROT-induced synucleinopathy (*P* < 0.001). Both metformin (20.85 ± 1.655) and empagliflozin (28.86 ± 1.601) significantly attenuated alpha-synuclein accumulation relative to the ROT group (*P* < 0.001), with metformin exerting a stronger effect than empagliflozin (*P* < 0.001). The combination therapy achieved the greatest reduction in alpha-synuclein burden (8.326 ± 0.8946), significantly surpassing the effects of both monotherapies (*P* < 0.001) (Figs. 6 and 7).

### Striatal dopamine levels

Metformin preserved dopamine levels more effectively (642.3 ± 52.3 pg/mL; *P* < 0.001 vs. ROT) than empagliflozin (460.2 ± 94.25 pg/mL; *P* < 0.001 vs. ROT), with a significant difference between the two treatments (*P* < 0.001). Combination therapy produced the highest dopamine levels (723.9 ± 68.7 pg/mL), which were significantly greater than those in the ROT and empagliflozin groups (*P* < 0.001) and comparable to the metformin group (*P* > 0.05) (Table 2).

**Table 2:** Comparison of striatal dopamine levels (pg/mL) measured by ELISA among the five groups.

	Control	ROT	MET + ROT	EMPA + ROT	MET+EMPA + ROT
Striatal dopamine levels (pg/mL) Mean ± S.D	833.4± 89.34	178.0± 41.43	642.3± 52.30	460.2± 94.25	723.9± 68.70
P1 P2 P3 P4		*P* < 0.001	*P* < 0.001 *P* < 0.001	*P* < 0.001 *P* < 0.001 *P* < 0.001	*P* < 0.05 *P* < 0.001 *P* > 0.05 *P* < 0.001

Each group consisted of 10 rats (*n* = 10). P1: comparison with the control group; P2: comparison with the diseased (ROT) group; P3: comparison with the MET + ROT group; P4: comparison with the EMPA + ROT group. A P-value ≤ 0.05 was considered statistically significant, while *P* ≤ 0.001 was considered highly significant. Data are expressed as mean ± standard deviation (SD).

### Assessment of PI3K and phosphorylated PI3K total protein levels by immunoblotting

**Fig. 8:**
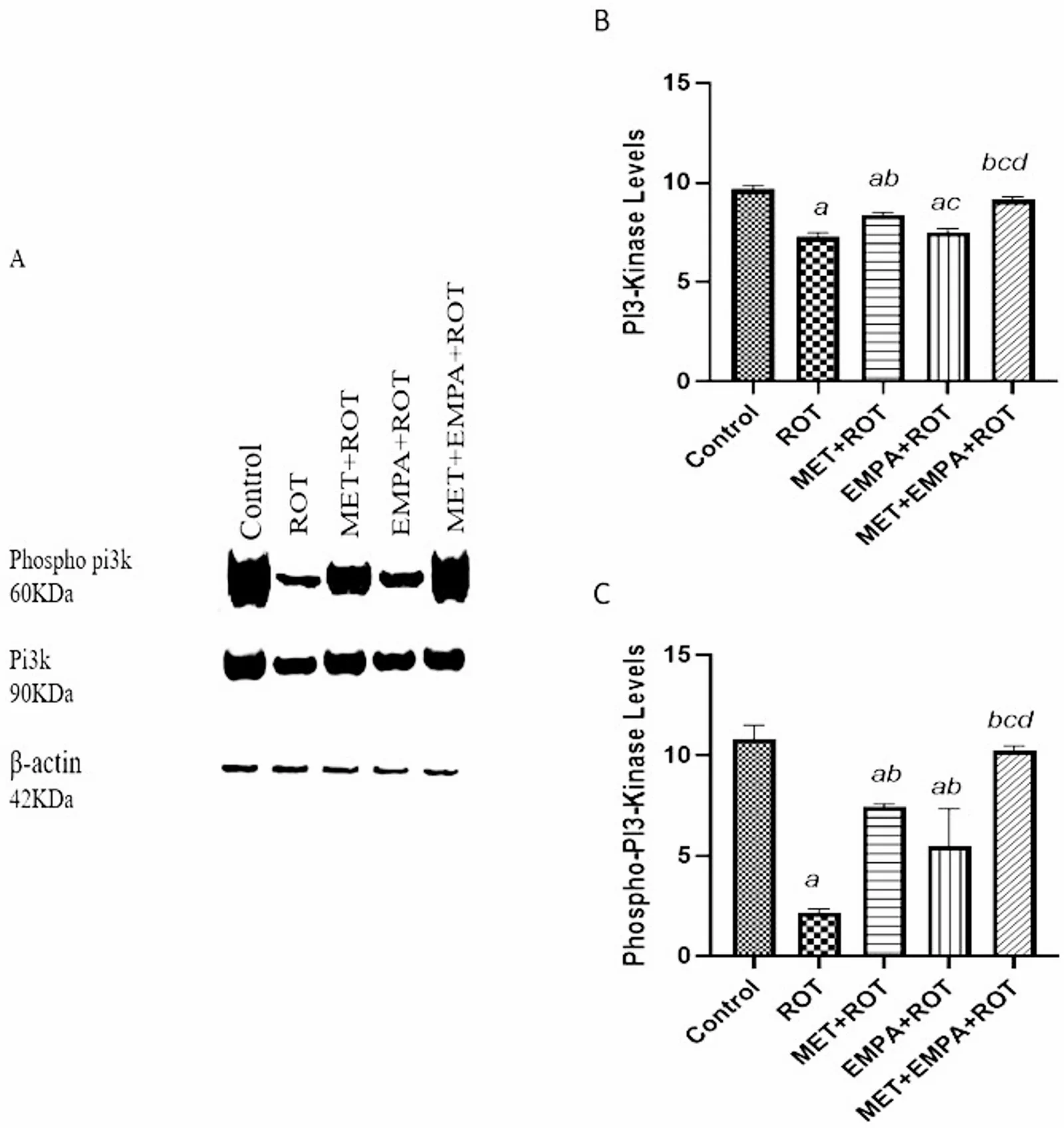
(**A**) Cropped Western blot images showing protein expression levels of phosphorylated phosphoinositide 3-kinase (p-PI3K; active form), total phosphoinositide 3-kinase (PI3K), and β-actin (loading control) across the different experimental groups. Full-length, uncropped blots are provided in Supplementary File 3. (**B**) PI3-kinase (PI3K) levels quantified by Western blot from brain homogenates; mean ± SD with significance annotations identical to Fig. 2B. (*n* = 3 per group). (**C**) Phosphorylated PI3-kinase (p-PI3K) levels quantified; data mean ± SD, significance as in (B) (*n* = 3 per group).

ROT significantly abrogated PI3K protein expression (7.25 ± 0.25) compared with the control group (9.65 ± 0.21; *P* < 0.001). Metformin treatment significantly restored PI3K levels (8.37 ± 0.15; *P* < 0.001 vs. ROT), whereas empagliflozin induced only a modest, non-significant increase (7.52 ± 0.18). Remarkably, combined therapy restored PI3K expression (9.15 ± 0.14) to baseline values, an effect that was significantly superior to monotherapies (*P* < 0.001) and indistinguishable from controls (Fig. 8) (Supplementary File 3).

Phospho-PI3K (a definitive biomarker for the activation state of the PI3K/AKT signaling cascade, a principal pathway governing neuronal survival) displayed a similar pattern: ROT markedly downregulated expression (2.21 ± 0.17 vs. 10.85 ± 0.69; *P* < 0.001). Both metformin and empagliflozin increased phospho-PI3K levels (7.44 ± 0.15 and 5.48 ± 1.87; *P* < 0.001 vs. ROT), while the combined regimen (10.24 ± 0.24) restored levels to near control (*P* > 0.05 vs. control), significantly outperforming the individual treatments (*P* < 0.05–0.001) (Fig. 8) (Supplementary File 3).

### Assessment of PPAR-γ gene expression by quantitative real-time PCR

The PPAR-γ gene expression was markedly suppressed by ROT (0.33 ± 0.05) compared with controls (1.00 ± 0.00; *P* < 0.001). Metformin significantly restored expression (0.59 ± 0.10; *P* < 0.001 vs. ROT) and produced higher levels than empagliflozin (0.46 ± 0.10; *P* < 0.05). Notably, the combination therapy elicited the greatest upregulation (0.75 ± 0.10; *P* < 0.001 vs. monotherapies), approaching control levels and indicating an enhanced effect on PPAR-γ–related pathways compared with either treatment alone (Table 3).

**Table 3:** Comparison of PPAR-γ expression levels measured by PCR among the five groups.

	Control	ROT	MET + ROT	EMPA + ROT	MET+EMPA + ROT
PPAR-γ Expression Levels Mean ± S.D	1.000 ± 0	0.3300 ± 0.04830	0.5900 ± 0.09944	0.4600 ± 0.09661	0.7500 ± 0.09718
P1 P2 P3 P4		*P* < 0.001	*P* < 0.001 *P* < 0.001	*P* < 0.001 *P* < 0.05 *P* < 0.05	*P* < 0.001 *P* < 0.001 *P* < 0.001 *P* < 0.001

Each group consisted of 10 rats (*n* = 10). P1: comparison with the control group; P2: comparison with the diseased (ROT) group; P3: comparison with the MET + ROT group; P4: comparison with the EMPA + ROT group. A P-value ≤ 0.05 was considered statistically significant, while *P* ≤ 0.001 was considered highly significant. Data are expressed as mean ± standard deviation (SD).

## Discussion

Parkinsonism is the second most prevalent neurodegenerative disorder after Alzheimer’s disease and is characterized by a progressive loss of dopaminergic neurons within the substantia nigra pars compacta (SNc), resulting in both motor and non-motor impairments^21,22^. Accumulating evidence indicates that Parkinsonism is driven by excessive inflammatory and oxidative mediators, which generate a sustained oxidative burden that triggers mitochondrial and endoplasmic reticulum (ER) stress^25,26^. These pathological processes promote α-synuclein aggregation and progressive dopaminergic neuronal loss, thereby recapitulating key features of Parkinsonian pathology^24^. In this context, metformin and empagliflozin have attracted growing interest due to their well-documented anti-inflammatory and antioxidant properties, which may counteract oxidative stress, attenuate neuroinflammation, and ultimately confer neuroprotection in Parkinsonism^38,39^.

In our work, we used ROT to reproduce Parkinsonism-like pathology in our experimental model. The rotenone-induced rat model reliably reproduces the neuropathological and biochemical hallmarks of Parkinsonism^40^, as ROT acts as a potent mitochondrial complex I inhibitor that disrupts oxidative phosphorylation, depletes ATP^41^, and promotes excessive production of reactive oxygen species (ROS). This oxidative burden triggers mitochondrial and endoplasmic reticulum (ER) stress^42,43^, leading to α-synuclein aggregation and dopaminergic neuronal loss, thus mimicking Parkinsonian pathology^41^.

The present study was designed to evaluate the neuroprotective potential of the antidiabetic agents empagliflozin and metformin, individually and in combination, against rotenone-induced neurodegeneration in rats. To our knowledge, this is the first study to explore the combined therapeutic efficacy of these two metabolic modulators in a rotenone-induced Parkinson’s disease model.

Drug repositioning has recently emerged as a promising and efficient strategy for developing treatments for neurodegenerative disorders. Among the repurposed candidates, the antidiabetic agents metformin and empagliflozin have demonstrated notable neuroprotective potential^9^, largely attributed to their antioxidant and anti-inflammatory properties mediated through modulation of intracellular signaling pathways^10^.

In neurodegenerative models, metformin and empagliflozin exhibit complementary properties supporting neuroprotection. Metformin has 50–60% oral bioavailability, a 4–9 h half-life, and limited blood–brain barrier penetration (10–20%) via organic cation transporters, with brain levels detectable within minutes. It reduces oxidative stress and improves mitochondrial function via AMPK activation^44,45^.

Empagliflozin, an SGLT2 inhibitor, has rapid absorption (Tmax ~ 1.5 h), a 12-hour half-life, and exerts indirect central effects through anti-inflammatory, antioxidant, and anti-apoptotic mechanisms^46^. No significant pharmacokinetic interaction occurs between the two drugs, supporting their combined use^47^.

In humans, the metformin–empagliflozin combination is extensively used in type 2 diabetes and is effective and well-tolerated. It reduces HbA1c by 0.5–0.6% beyond placebo, along with weight loss, blood pressure reduction, and cardiovascular benefits. The combination has a low hypoglycemia risk when used without insulin or sulfonylureas^47^.

Common adverse effects include GI issues (metformin) and genitourinary infections (empagliflozin); rare serious events include lactic acidosis and ketoacidosis. Contraindicated in moderate-to-severe renal impairment. Minimal pharmacokinetic interactions, but caution with insulin/sulfonylureas (hypoglycemia) or diuretics (dehydration). Complementary mechanisms support safe, effective neuroprotective potential^48^.

Male rats were selected as experimental subjects in this study. Standard practice in preclinical research often favors the use of male rats and mice, primarily due to logistical considerations, including reduced costs and simplicity of experimental control, compared to female counterparts. Female rodents undergo a four- to five-day estrous cycle, analogous to a compressed human menstrual cycle, resulting in significant hormonal fluctuations that can introduce variability across experimental groups^49^.

Hormonal state-dependent physiological responses may confound results and hinder reproducibility if subjects are not synchronized or adequately monitored. As previously described, reliable data collection with female rodents often requires daily vaginal cytology to determine the stage of the estrous cycle, especially in studies sensitive to endocrine variation. Without such control, outcome measurements may become inconsistent. Furthermore, up to four times more female animals may be necessary to ensure the synchronization of cycles within groups, resulting in increased procedural complexity, resource demand, and costs. Even with these precautions, data may exhibit greater variability, complicating data interpretation and potentially limiting publishability. These factors, coupled with funding constraints, are widely cited as reasons for the predominant use of male rodents in experimental designs, as detailed in existing literature^49^.

In the present study, treatment with metformin or empagliflozin significantly enhanced locomotor activity in the open-field test, as well as grip strength and balance in the static rod and inverted grid tests. Notably, their combined administration (MET+EMPA) produced the most pronounced functional recovery, with performance approaching control values. This enhanced behavioral improvement suggests complementary neuroprotective actions, potentially involving mitochondrial stabilization and reinforcement of antioxidant defenses.

These behavioral outcomes are consistent with previous investigations reporting the anti-Parkinsonian efficacy of empagliflozin. Motawi et al.,^5^ Mousa et al.,^6^ Ahmed et al.,^8^ and Mohammed et al.^38^ demonstrated that empagliflozin significantly alleviates rotenone-induced locomotor and neuromuscular impairments, normalizes gait parameters, and preserves dopaminergic neuronal integrity within the SNc and striatum.

Complementing these mechanistic findings, Salem et al.^50^ demonstrated that ROT administration in diabetic rats induced marked metabolic, neurobehavioral, biochemical, and histopathological abnormalities that closely mimicked Parkinsonian pathology, with the diabetic state further exacerbating neurodegenerative outcomes. Treatment with semaglutide or metformin significantly ameliorated these behavioral impairments, while combined therapy most effectively restored motor coordination, exploratory activity, and olfactory discrimination—indicating a synergistic neurobehavioral benefit of dual metabolic modulation.

Dopamine, produced in the nigrostriatal pathway (SNc → dorsal striatum), is a critical regulator of voluntary movement through basal ganglia modulation^51^. In Parkinson’s disease (PD) and experimental models such as rotenone-induced Parkinsonism in rats, degeneration of nigrostriatal neurons causes marked striatal dopamine depletion, leading to motor deficits, including bradykinesia and impaired coordination^52^.

Conversely, restoring striatal dopamine levels re-establishes normal basal ganglia function. A well-documented causal relationship exists between striatal dopamine content and motor performance, with strong correlations reported in both clinical and preclinical studies. Thus, striatal dopamine concentration serves as a direct neurochemical correlate of motor function, and its restoration following treatment reliably predicts functional motor recovery^53^.

In our study, the striatal dopamine levels, which were markedly depleted in rotenone-treated rats, were significantly restored by both monotherapies—most effectively in the combination group—indicating preservation of nigrostriatal integrity. Consistent with our observations, previous studies have reported a marked reduction in striatal dopamine levels in combined diabetes and Parkinson’s disease (DM + PD) models compared with controls, whereas treatment with antidiabetic agents such as semaglutide and metformin significantly restored dopamine content, with the most pronounced improvement observed following combination therapy^27^.

Similarly, several investigations have demonstrated that empagliflozin treatment significantly restored dopamine turnover and preserved dopaminergic neurotransmission relative to ROT exposure, although dose-dependent variations in efficacy have been reported^8,38^. Collectively, these findings support a neuroprotective role for both metformin and empagliflozin in Parkinsonian pathology, likely mediated through complementary mechanisms involving attenuation of oxidative stress, suppression of neuroinflammatory signaling, modulation of astrocytic dysfunction, and preservation of nigrostriatal dopaminergic integrity, in strong agreement with the outcomes of the present study.

Histopathological analysis in our study revealed pronounced neuronal degeneration, pyknosis, and cytoplasmic vacuolation within the substantia nigra pars compacta (SNc) and putamen of rotenone-treated rats, consistent with classical Parkinsonian pathology. Treatment with either metformin or empagliflozin significantly attenuated neuronal loss and cytoplasmic eosinophilia, while their combined administration showed marked improvement in neuronal morphology, indicating an additive neuroprotective effect.

Immunohistochemical analysis showed a pronounced increase in α-synuclein immunoreactivity following ROT exposure, which was markedly attenuated by all treatment regimens, particularly the combination therapy. These results confirm that co-treatment effectively mitigates protein aggregation and neuronal degeneration.

These findings are consistent with Motawi et al.^5^ and Mousa et al.,^6^ who reported that empagliflozin preserved nigral cytoarchitecture, decreased neuronal pyknosis, and suppressed glial activation. Similarly, Ahmed et al.^8^ demonstrated that empagliflozin reduced cerebellar damage and restored normal architecture, emphasizing its tissue-protective effects across brain regions. Collectively, our histological and immunohistochemical observations substantiate the superior neuroprotective efficacy of combined therapy through coordinated suppression of oxidative stress, inflammation, and α-synuclein accumulation.

Salem et al.^50^ reported that histopathological examination revealed extensive neuronal degeneration, prominent Lewy body deposition, hemorrhagic foci, and marked vacuolation in the diabetic Parkinsonian group, particularly within the substantia nigra and hippocampal regions.

These pathological alterations were substantially alleviated in the treated cohorts, with the semaglutide–metformin combination demonstrating near-complete restoration of normal cytoarchitecture, minimal pyknotic changes, and reduced vascular congestion.

Emerging evidence suggests that PPAR-γ signaling plays a multifaceted neuroprotective role in neurodegenerative disorders, extending beyond metabolic regulation to include modulation of neuroinflammation, oxidative stress, mitochondrial dysfunction, and synaptic integrity. Activation of PPAR-γ has been shown to suppress pro-inflammatory cytokine production, inhibit microglial activation, and enhance antioxidant defenses, thereby contributing to neuronal survival in Parkinsonian models. In addition, recent studies indicate that PPAR-γ signaling is closely linked to synaptic plasticity, a critical process underlying motor and cognitive function. PPAR-γ activation may regulate the expression of synaptic proteins, improve neuronal connectivity, and preserve dopaminergic neurotransmission, thereby ameliorating behavioral deficits associated with neurodegeneration^54^.

In the present study, the observed upregulation of PPAR-γ expression following metformin and empagliflozin treatment may therefore represent an important mechanistic link between metabolic modulation and neuroprotection. The behavioral improvements observed in treated animals could be partially attributed to PPAR-γ–dependent enhancement of synaptic plasticity and neuronal resilience in addition to the anti-inflammatory and antioxidant effects identified in this study. These findings are supported by recent evidence demonstrating that PPAR-γ signaling contributes to the regulation of synaptic function and neural plasticity in neurodegenerative diseases. Collectively, these observations further support the potential therapeutic relevance of targeting PPAR-γ pathways in Parkinsonism and related neurodegenerative conditions^54^.

The PI3K/AKT pathway critically regulates neuronal survival, apoptosis, and inflammation in Parkinsonism models. Its suppression drives dopaminergic degeneration via oxidative stress and pro-apoptotic cascades, while activation confers neuroprotection by inhibiting apoptosis, reducing oxidative stress, suppressing neuroinflammation, and enhancing autophagy^55^.

In this study, we investigated the PI3K/AKT and PPAR-γ signaling pathways. Both metformin and empagliflozin restored the PI3K/AKT signaling pathway, as evidenced by a significant increase in both total and phosphorylated PI3K levels. Moreover, their combination nearly normalized PI3K/AKT activity, suggesting enhanced activation of pro-survival signaling pathways. The PI3K/AKT pathway plays a fundamental role in neuronal survival, metabolism, and defense against neurodegeneration in Parkinsonism. Activation of PI3K/AKT promotes neuronal viability by inhibiting pro-apoptotic factors and regulating downstream targets such as glycogen synthase kinase-3β (GSK3β). Inactivation of GSK3β mitigates α-synuclein aggregation and tau hyperphosphorylation—two key pathogenic processes in Parkinsonism^56^.

Moreover, the PI3K/AKT pathway modulates neuroinflammatory responses by influencing microglial and astrocytic phenotypes, thereby shifting them toward neuroprotective states^57^.

Peroxisome proliferator-activated receptor gamma (PPAR-γ) constitutes another critical mediator of neuroprotection, exerting anti-inflammatory effects, enhancing mitochondrial function, and reducing oxidative stress within dopaminergic neurons^58^.

Agonists of PPAR-γ, including thiazolidinediones, have demonstrated neuroprotective efficacy in Parkinsonism models by attenuating microgliosis and modulating mitochondrial bioenergetics, suggesting that therapeutic targeting of these pathways may attenuate Parkinsonism progression^59^.

Importantly, crosstalk between the PI3K/AKT and PPAR-γ signaling cascades may synergistically strengthen neuronal resilience by coordinately regulating survival mechanisms, inflammatory modulation, and metabolic homeostasis. This interplay provides a rationale for combination therapies targeting both pathways in Parkinsonism treatment^60^.

The observed increase in PPAR-γ expression following treatment further supports the mechanistic interplay between metabolic and neuroprotective pathways. PPAR-γ activation is known to modulate mitochondrial function, inhibit oxidative stress, and cross-regulate PI3K/AKT signaling. Thus, its upregulation likely contributed to the observed improvement in dopaminergic survival and functional outcomes.

The current findings align with several reports highlighting the neuroprotective potential of antidiabetic agents in Parkinsonism. Empagliflozin has been shown by Motawi et al.,^5^ Mousa et al.,^6^ and Ahmed et al.^8^ to mitigate ER stress, oxidative injury, and inflammation in ROT models through normalization of the GRP78/PERK/eIF2α/CHOP pathway, suppression of lipid peroxidation, and restoration of antioxidant reserves (GSH, SOD). Moreover, it activates neurotrophic and survival mediators, including BDNF, CREB, and phosphorylated Akt, underscoring its capacity to preserve dopaminergic function and inhibit α-synuclein aggregation. Histopathological evidence from these studies further supports empagliflozin’s role in maintaining neuronal architecture and attenuating glial activation, as demonstrated by reduced GFAP and Iba1 immunoreactivity.

Similarly, metformin exhibits multifaceted neuroprotection in experimental Parkinsonism. Katila et al.^61^ demonstrated that metformin reduces rotenone-induced cytotoxicity, ROS accumulation, and caspase-3/7 activation via AMPK-dependent mechanisms. It restored GSH and SOD activity, preserved mitochondrial complex I function, and increased PGC-1α expression, implicating stimulation of mitochondrial biogenesis. Importantly, the study revealed that metformin’s effects are mediated through AMPK- and AKT-dependent Nrf2/HO-1 activation, linking metabolic regulation to redox homeostasis. Complementary studies by Wang et al.^62^ and Khadija Tayara et al.^63^ further showed that metformin suppresses astrocytic senescence, inhibits microglial activation, and reduces proinflammatory mediators (IL-1β, TNF-α), collectively mitigating neuroinflammation and glial toxicity.

The consistency between our data and these findings strongly supports the hypothesis that empagliflozin and metformin act through convergent yet complementary mechanisms—empagliflozin primarily via PPAR-γ and PI3K/AKT activation, and metformin via AMPK/Nrf2/HO-1 signaling—to preserve mitochondrial and redox homeostasis. Their combined use therefore amplifies neuroprotective signaling, reduces α-synuclein aggregation, and restores dopaminergic function more effectively than monotherapy.

The novelty of the present work lies in demonstrating, for the first time, the enhanced neuroprotective effects of combined metformin and empagliflozin therapy in a rotenone-induced Parkinsonism model. Mechanistically, the combination may exert complementary actions through modulation of PPAR-γ and PI3K/AKT signaling pathways, preservation of mitochondrial function, and attenuation of α-synuclein aggregation. This dual metabolic–neuroprotective approach helps bridge the link between antidiabetic therapeutics and neurodegeneration, supporting the potential relevance of metabolic modulation as a therapeutic strategy in Parkinsonism.

### The limitations of the study and future perspectives

Several limitations of the present study should be acknowledged. The study employed a short-term rotenone-induced Parkinsonism model using only male rats, which limits extrapolation to the progressive nature of human Parkinson’s disease and precludes evaluation of sex-dependent treatment responses. Mechanistically, the analysis was restricted to PI3K/p-PI3K protein expression and PPAR-γ mRNA levels, without assessment of downstream PI3K/AKT signaling molecules, PPAR-γ protein expression, oxidative stress, neuroinflammatory, mitochondrial, autophagy, or apoptosis-related markers, nor were pharmacological pathway inhibition studies performed. Therefore, the proposed molecular mechanisms should be considered associative rather than causal.

Histopathological and immunohistochemical analyses were semi-quantitative and may be subject to observer variability. Although α-synuclein immunohistochemistry was performed, biochemical characterization of pathological α-synuclein species was not conducted. Similarly, dopaminergic neuronal preservation was not confirmed using tyrosine hydroxylase immunostaining or other complementary neuronal markers. Dopamine was measured by ELISA in whole-striatal homogenates without regional analysis or HPLC validation.

In addition, Western blot and qPCR analyses lacked comprehensive validation, including full MIQE-compliant reporting and downstream protein confirmation. Commercial tablet formulations were used without an excipient-control group, while pharmacokinetic profiling, brain drug exposure, dose optimization, and systemic metabolic assessments were not performed. Furthermore, formal pharmacological synergy analyses were not conducted, limiting definitive interpretation of drug interactions. Finally, although appropriate statistical analyses were applied, effect sizes, confidence intervals, and comprehensive multiplicity correction across all endpoints were not included.

Future studies incorporating chronic and genetically relevant Parkinson’s disease models, both sexes, comprehensive molecular validation, pharmacokinetic and metabolic assessments, HPLC-based dopamine quantification, and formal synergy analyses will further strengthen the mechanistic understanding and translational relevance of the combined neuroprotective effects of empagliflozin and metformin.

## Conclusion

This study demonstrates that both metformin and empagliflozin exert significant neuroprotective effects against rotenone-induced neurodegeneration in rats, improving motor function, preserving dopaminergic integrity, and attenuating α-synuclein pathology. Importantly, combined treatment with these agents produced superior outcomes across behavioral, biochemical, and histopathological parameters compared with monotherapies. Mechanistically, the enhanced effects observed with the combination therapy may involve restoration of PI3K/AKT signaling, upregulation of PPAR-γ expression, and attenuation of oxidative stress and protein aggregation. These findings highlight the therapeutic potential of repurposing metabolic modulators for Parkinson’s disease and underscore the benefit of dual target approaches in neurodegenerative disorders.

## Supplementary Information

Below is the link to the electronic supplementary material.

Supplementary Material 1

Supplementary Material 2

Supplementary Material 3

Supplementary Material 4

Supplementary Material 5

Supplementary Material 6

Supplementary Material 7

## Data Availability

The datasets generated and analyzed during the current study are available from the corresponding author upon reasonable request.
