# Supplementary material for: Neuroprotective effects of empagliflozin and metformin combined therapy on rotenone induced Parkinsonism in Wistar rats via PI3K and PPARγ modulation
Source: Sci Rep. 2026 Sep 8;16:28103. doi: 10.1038/s41598-026-68289-w (PMC13554164; doi:10.1038/s41598-026-68289-w)
Supplement: Supplementary file 1 [file 41598_2026_68289_MOESM1_ESM.docx]

**ARRIVE 2.0 Essential 10 Checklist**

| **ARRIVE Item** | **Manuscript location** |
| --- | --- |
| 1. Study design: groups, experimental unit, and overall design described. | Methods: Experimental Design and Sample Size, pp. 7–8. |
| 2. Sample size: total animals and animals per group reported; a priori power analysis provided. | Methods: Experimental Design and Sample Size, p. 8. |
| 3. Inclusion and exclusion criteria Statement that no animals or data points were excluded and no outlier exclusion criteria were applied. | Methods: Behavioral Assessment, p. 8. |
| 4. Randomisation: Computer-generated random allocation described. | Methods: Experimental Design and Sample Size, p. 8. |
| 5. Blinding: Single-blinded outcome assessment reported for behavioral, biochemical, and histological analyses. | Methods: Experimental Design and Sample Size, p. 8. |
| 6. Outcome measures: Primary behavioral, histological, biochemical, and molecular outcomes defined. | Methods: Behavioral Assessment; Histopathology and Immunohistochemistry; Biochemical Analysis; Western Blotting; qPCR, pp. 8–13. |
| 7. Statistical methods: tests used, assumption checks, post hoc procedures, and significance threshold reported. | Methods: Data and Statistical Analysis, p. 13. |
| 8. Experimental animals: species, strain, sex, age, weight, and source described. | Methods: Experimental Animals, p. 6. |
| 9. Experimental procedures: Drug preparation, dosing, route, timing, behavioral testing, tissue collection, and molecular procedures described in sufficient detail. | Methods: Drug Preparation through qPCR Analysis, pp. 7–13. |
| 10. Results: Summary statistics and group comparisons reported for all major outcomes. | Results section, pp. 14–18. |

**ARRIVE 2.0 Recommended Set (summary)**

| **Item** | **Manuscript location** |
| --- | --- |
| Abstract | Abstract, p. 3. |
| Background | Introduction, pp. 4–5. |
| Objectives | Introduction, p. 5. |
| Ethical statement | Methods: Ethical Statement, p. 6; Declarations, p. 29. |
| Housing and husbandry | Methods: Experimental Animals, p. 6. |
| Animal care and monitoring | Methods: Drug Preparation (daily monitoring) and Tissue Collection (humane euthanasia), pp. 7 and 9. |
| Interpretation/scientific implications | Discussion, pp. 19–27. |
| Generalisability/translation | Discussion and Limitations, pp. 19–27. |
| Protocol registration | Not applicable (no preregistered protocol). |
| Data access | Data Availability, p. 2 and p. 29. |
| Declaration of interests | Competing Interests, p. 2 and p. 29. |
