# Supplementary material for: Neuroprotective effects of empagliflozin and metformin combined therapy on rotenone induced Parkinsonism in Wistar rats via PI3K and PPARγ modulation
Source: Sci Rep. 2026 Sep 8;16:28103. doi: 10.1038/s41598-026-68289-w (PMC13554164; doi:10.1038/s41598-026-68289-w)
Supplement: Supplementary file 2 [file 41598_2026_68289_MOESM2_ESM.docx]

Fig 1. Brain section of substantia nigra pars compacta of control animal G1 showing normal pyramidal neuronal cells with normal cytoplasm and normal vesiclular nucleus (arrowheads), H&E stain, bar= 50 µm.

Fig 2. Brain section of substantia nigra pars compacta of ROT group G2 showing severe degree of degenerative ischemic injury of the neuronal cells (black arrowheads) associated with marked gliosis (black arrow) and congestion of blood capillaries and haemorrhage (yellow arrow), H&E stain, bar= 50 µm.

Fig3 Brain section of substantia nigra pars compacta of ROT group G2 showing severe degree of ischemic neuronal injury of the neuronal cells (black arrowheads) associated with atrophy of the neurons, basophilic clumping of Nissl’s granules, and marked gliosis (black arrow) (yellow arrow), H&E stain, bar = 50 µm.

Fig4. Brain section of substantia nigra pars compacta of MET+ROT group G3 showing decrease the ischemic changes within the neuronal cells (arrowhead) with cellular vacuolation of some neurons, and decrease both gliosis and neuronophagia (black arrow) (white arrowhead indicates normal neurons), H&E stain, bar = 50 µm.

Fig5. Brain section of substantia nigra pars compacta of EMPA+ROT group G4 showing decrease ischemic features within the neuronal cells (black arrowhead) with mild gliosis (black arrow) and with noticeable increase number of normal neurons (white arrowhead), H&E stain, bar= 50 µm.

Fig6. Brain section of substantia nigra pars compacta of MET +EMPA +ROT group G5 showing marked decrease ischemic neuronal cells associated with decrease neuronophagia (black arrowhead) and with remarkable increase of the number of normal neurons (white arrowhead), H&E stain, bar = 50 µm.

Fig7. Brain section of putamen region of control group G1 showing normal neuronal cells (arrowhead) and with normal striatal fibers (arrow), H&E stain, bar = 50 µm.

Fig8. Brain section of putamen region of ROT group G2 showing severe degree of ischemic degenerative changes within the neuronal cells with marked nuclear pyknosis (white arrowhead) and axonal degenerative changes associated with disruption of the striatal fibers (black arrowhead), H&E stain, bar = 50 µm.

Fig9 . Brain section of putamen region of ROT group G2 showing severe loss of the neuronal cells (white arrowhead) associated with severe disruption of the striatal fibers (black arrowhead), H&E stain, bar= 50 µm.

Fig10 . Brain section of putamen region of MET +ROT group G3 perineuronal oedema (white arrowhead) and vacuolation of the bundles of the striatal fibers (black arrowhead), H&E stain, bar= 50 µm.

Fig11. Brain section of putamen region of EMPA+ROT group G4 showing decrease basophilic neuronal cells (white arrowhead) and with normal striatal fibers (black arrowhead), H&E stain, bar= 50 µm.

Fig12. Brain section of putamen region of MET +EMPA+ROT group G5 showing mild degree of perineuronal vacuolation (black arrowhead) and with normal striatal fibers (black arrowhead), H&E stain, bar= 50 µm.
