# Supplementary material for: Neuroprotective effects of empagliflozin and metformin combined therapy on rotenone induced Parkinsonism in Wistar rats via PI3K and PPARγ modulation
Source: Sci Rep. 2026 Sep 8;16:28103. doi: 10.1038/s41598-026-68289-w (PMC13554164; doi:10.1038/s41598-026-68289-w)
Supplement: Supplementary file 3 [file 41598_2026_68289_MOESM3_ESM.docx]

**1-phospho pi3k**

Experiment 1 :Anti- Phospho-PI3 Kinase p85Antibody expression level for samples

Experiment 1: Calculation of Anti- Phospho-PI3 Kinase p85form molecular weight for samples.

Experiment 2: Anti-Phospho-PI3 Kinase p85Antibody  expression level for samples.

Experiment 2: calculation of Anti- Phospho-PI3 Kinase p85form molecular weight for samples.

Experiment 3: Anti- Phospho-PI3 Kinase p85Antibody expression level for samples.

Experiment 3: calculation of Anti- Phospho-PI3 Kinase p85form molecular weight for samples.

**2- PI3 Kinase**

Experiment 1: Anti- PI3 Kinase p85 Antibody expression level for samples.

Experiment 1: Calculation of Anti- PI3 Kinase p85 form molecular weight for samples.

Experiment 2: Anti- PI3 Kinase p85 Antibody expression level for samples.

Experiment 2: calculation of Anti- PI3 Kinase p85 form molecular weight for samples.

Experiment 3: Anti- PI3 Kinase p85 Antibody expression level for samples.

Experiment 3: calculation of Anti- PI3 Kinase p85 form molecular weight for samples.
