# Supplementary material for: Neuroprotective effects of empagliflozin and metformin combined therapy on rotenone induced Parkinsonism in Wistar rats via PI3K and PPARγ modulation
Source: Sci Rep. 2026 Sep 8;16:28103. doi: 10.1038/s41598-026-68289-w (PMC13554164; doi:10.1038/s41598-026-68289-w)
Supplement: Supplementary file 4 [file 41598_2026_68289_MOESM7_ESM.docx]

**Supplementary Data:**

**Supplementary Video 1:** Representative Open-Field Test Videos for All Experimental Groups.

**Supplementary Video 2:** Representative videos of the static rod test demonstrating behavioral impairments in rotenone-treated rats compared with the ROT and control groups.

**Supplementary Video 3:** Representative videos of the inverted grid test demonstrating motor deficits in rotenone-treated rats compared with the ROT and control groups.

**Supplementary File 1:** ARRIVE 2.0 Checklist

**Supplementary File 2:** Original, non-magnified histological images of the substantia nigra pars compacta and putamen from all experimental groups.

**Supplementary File 3:** Full-length Western blot images obtained from three independent experiments.
